# A Bill Enabling Her Majesty to Grant New Charters to Certain Colleges of Physicians and Surgeons

**Published:** 1845-04

**Authors:** 


### Art. XVIII.

*A Bill for enabling Her Majesty to grant New Charters to certain Colleges of Physicians and Surgeons. Ordered to be printed 25th Feb.* 1845.

This Bill enables Her Majesty to grant new charters to the Colleges of Physicians of London, Edinburgh, and Dublin, and to the College of Surgeons of Edinburgh. It gives no information as to the character of the charters to be granted; and as none of these have been as yet laid before parliament, the profession generally must, for a time, remain ignorant of their respective provisions. The only one of the intended charters, of the contents of which we possess any knowledge, is that of the College of Physicians of London; and it is with great concern—we may indeed say, as a member of that body—it is with no slight feeling of shame, that we announce to our brethren beyond the pale of the college, that it contains one most important clause which, in our humble opinion, not only vitiates all that is good in it, (and it contains much that is good,) but stamps it as a charter, as in the highest degree illiberal and unjust, alike unsuited to the spirit of the present time, and unworthy of the institution whence it emanates. The provision to which we refer is *the limitation of the fellowship to a small number of the members of the corporation.* At the moment we are writing, it is not finally decided whether the number of FELLOWS shall be definitively limited to *two hundred,* or shall bear a fixed proportion to the varying number of Licentiates or Associates. But, in either case, the result will be the same, viz. that *only a small proportion of the* MEMBERS (one fourth, one fifth, or one sixth) *can by possibility become* FELLOWS, that is to say, *can obtain any corporate privileges or attain any of the offices or honours of the College!* When it is considered that by the new charter, the College is reconstituted as a CORPORATION; that its title is changed to that of the ROYAL COLLEGE

of Physicians in England; that by the new Bill every future English physician is *compelled* to join the corporation as a *member*, and to pay a fee on joining it; and yet that the obtaining of any corporate privileges or attaining any corporate honours, can only, by possibility, fall to the lot of a small minority;—it can hardly be imagined that such a constitution could have been framed at the present day by the members of a scientific body, or, if framed, that it could obtain the sanction of the government. That it has been framed, however, is certain; but we are far from believing that it will obtain the sanction of government without modification.

The charter recently granted to the College of Surgeons, and which has excited so much discontent in that branch of the profession, is greatly more liberal and just than the one under consideration; inasmuch, as by the surgeons' charter, *every member may become a* fellow, and thus attain all corporate rights and privileges, on submitting to certain conditions therein laid down. It remains to be seen in what light the Physicians will regard the charter about to be granted to their College; but if, on being made aware of its character, they sit down in quiet and silent content, we shall—to say no more—be surprised. The college, however, in such a case, may be justified in considering its proceedings as sanctioned by those most affected by them; and though we will not the less maintain the justness of our own views, we will at once admit that we have been mistaken as to the opinions and feelings of the physicians of England.

Instead of stating, as we have done, that the future Members or Associates of the College of Physicians possess by the charter *no* corporate rights or privileges, we ought to have stated that *one* privilege is conceded, which the College may possibly regard as of some value: as, however, we consider it the reverse of valuable, we have rejected it from our category of corporate rights. The privilege is this: The members are permitted, on one day in the year, to meet the Fellows in the College, and to vote, *in conjunction with them*, in the election of Fellows (four or five, perhaps,) to fill up the few vacancies occasioned by death! For ourselves, we confess that we hope never to see a spectacle so melancholy—to use no harsher term—as that of a body of gentlemen thus consenting to compromise at once their dignity and their rights, by sanctioning an act of injustice towards the whole body of which they are members, and thus assisting in their own degradation.

In making these observations, we consider ourselves as discharging an imperative duty towards the College of Physicians. Considering the provision of the charter here animadverted on, as calculated to be still more injurious to the College than to the profession, we feel that we should not be fulfilling the solemn obligation we came under on becoming one of its members, if we failed to advocate any measures which we deem calculated to promote its best interests. The voice of the profession, if it chooses to speak, may be listened to, though our words may have been unheeded; while its silence, if it remains silent, will at least save the College from the annoyance of any more fruitless discussions within its own walls.

